# Focal Neurological Seizure due to Hyperglycemic Hyperosmolar Non-Ketotic Syndrome in Undiagnosed Diabetes Mellitus

**DOI:** 10.7759/cureus.9909

**Published:** 2020-08-21

**Authors:** Mihir Odak, Steven Douedi, Vandan Upadhyaya, Mustafa Fadhel, James Cosentino

**Affiliations:** 1 Internal Medicine, Jersey Shore University Medical Center, Neptune City, USA; 2 Pulmonary and Critical Care, Jersey Shore University Medical Center, Neptune City, USA

**Keywords:** diabetes, focal neurological seizures, hyperglycemic hyperosmolar non-ketotic syndrome, hyperglycemia

## Abstract

Hyperglycemic hyperosmolar non-ketotic syndrome (HHNS) is a life-threatening complication of type 2 diabetes mellitus with a wide range of presenting symptoms. Neurological symptoms, such as coma, can also be part of the manifestation of HHNS; however, focal seizures remain a rare but notable association.

A 85-year-old male patient with no history of diabetes presented to our emergency department complaining of a two-day history of twitching movements of his left wrist. Laboratory findings suggested HHNS and his hemoglobin A1c were found to be 10.2%. He was aggressively treated in the intensive care unit with fluids and insulin which also resolved his seizure episodes. He was ultimately discharged in stable condition without any seizure-like activity while having good glycemic control.

According to the American Diabetes Association, about 25% of all individuals 65 years and older have diabetes mellitus. With an increasing prevalence, the complications of uncontrolled diabetes are also becoming more notable. While the neurological deficits associated with HHNS are focal, the mechanism by which this occurs is still poorly understood and underreported warranting further studies.

## Introduction

Hyperglycemic hyperosmolar non-ketotic syndrome (HHNS) is an acute and severe complication of type 2 diabetes mellitus [1]. HHNS is characterized by dehydration, severe hyperglycemia, and possible neurological impairment, in the absence of ketosis. HHNS cases account for approximately 1% of all annual hospital admissions for diabetes-related conditions, and typically affect people aged 50 to 60 years [2]. HHNS is suggested when the initial glucose measurements are greater than 600 mg/dL, along with a plasma osmolarity greater than 320 mOsm/L, and an absence of ketosis [3]. We present a case of HHNS in a patient with undiagnosed type 2 diabetes mellitus and focal neurological seizure as the initial symptom. We hope to highlight HHNS as part of differential diagnosis in patients with focal neurological seizures.

## Case presentation

An 85-year-old male patient with a past medical history significant for hypertension, polycystic kidney disease, and hyperlipidemia, presented to the emergency department complaining of a two-day history of twitching movements of his left wrist. The patient reported that his twitching movements were localized to his left hand and wrist and would occur intermittently lasting for about one minute before abating. He reported no loss of consciousness, confusion, dizziness, incontinence of bowel or bladder, gait abnormalities, speech difficulties or tongue twitching, or changes in his vision. The patient’s review of symptoms was also negative for chest pain, shortness of breath, abdominal pain, fever, chills, nausea, vomiting, and diarrhea. The patient reported no polydipsia, but endorsed frequent urination. Since the patient had never experienced these symptoms before, he came to the emergency department

On presentation, patient’s vital signs were a blood pressure of 168/83 mmHg, heart rate of 77 beats per minute, temperature of 98.6 degrees Fahrenheit, and oxygen saturation of 98% on room air. The patient’s laboratory findings were suggestive of HHNS (Table 1). 

**Table 1:** Laboratory and imaging findings on presentation.

Labs/Imaging	Value	Reference Range
Venous pH	7.29	7.32–7.42
Hemoglobin A1C	10.2%	4.8%–5.6%
Sodium (Na)	125 mmol/L	136–135 mmol/L
Potassium (K)	4.7 mmol/L	3.5–5.2 mmol/L
Magnesium (Mg)	2.3 mg/dL	1.3–2.5 mg/dL
Calcium (Ca)	9.2 mg/dL	8.5–10.5 mg/dL
Albumin	3.8 g/dL	3.5–5.0 g/dL
Urinalysis	Glucose, large ketones: negative; protein: negative; blood: negative; bacteria: negative	Glucose: negative; ketones: negative; protein: negative; blood: negative; bacteria: negative
CT head	No intracranial pathology	-
MRI brain	No infarct or hemorrhage, findings most compatible with chronic microvascular ischemic disease	-

The patient was subsequently started on two liters of normal saline bolus along with a continuous insulin infusion and 20 mEq of potassium chloride in sterile water. The patient was transferred to the intensive care unit for treatment of a hyperglycemic hyperosmolar non-ketotic state in the setting of newly diagnosed diabetes mellitus. The patient was concurrently started on a continuous insulin infusion; as the insulin resolved the patient’s hyperglycemia, the patient’s seizure activity decreased in frequency, until resolved completely. After six hours on an insulin infusion and subsequent bridge to 18 units of lantus, the patient’s blood glucose level came down to 90 mmol/L. The patient was then stable for transfer to the inpatient floor teams for continued newly diagnosed diabetes mellitus type 2 management. He was discharged from the hospital two days later on 18 units of lantus, 1 milligram (mg) repaglinide three times a day with meals, and sitagliptin 50 mg before breakfast, with advice to follow up with outpatient endocrine and neurology services. He remained symptom free at one-month and six-month follow-ups with well-controlled glucose. 

## Discussion

The incidence of neurological dysfunction in HHNS cases currently stands at 10%-25% [4,5]. The mechanism by which neurological dysfunction occurs is yet unclear. A prevailing theory is that in the setting of profound hyperglycemia, the Krebs cycle is inhibited leading to depletion of gamma-aminobutyric acid (GABA) levels via increased metabolism, thereby lowering the seizure threshold [5]. Other possible explanations of seizures in HHNS include osmotic diuresis and dehydration, as well as hyponatremia, and hyperglycemic damage to cerebral vasculature causing transient ischemic disease [5]. Neurological deficits in HHNS are typically focal, and with improvement in the glycemic and osmolar status, the neurological deficit will likewise improve [6]. The neurological manifestations seen in HHNS include seizures, chorea, hemiballismus, epilepsia partialis continua (EPC), and coma, with coma being the most common [5]. Although coma is the most commonly seen neurological manifestation of HHNS, focal seizures and EPC should suggest a possible osmolar etiology for neurological dysfunction.

HHNS occurs due to an elevation of counter-regulatory hormones, including cortisol, glucagon, and catecholamines, which trigger gluconeogenesis, and this results in hyperglycemia and a hyperosmolar state [1]. When the excess glucose in the blood reaches the kidney and exceeds the transport maximum of the nephron for glucose, the patient experiences osmotic diuresis and profound dehydration [4]. Presentation of neurological symptoms, such as encephalopathy, hyperkinetic symptoms, or focal or partial seizures, in the setting of significant hyperglycemia (blood glucose greater than 600 mg/dL) should raise suspicion for HHNS, especially if subsequent neurological imaging reveals no abnormality and if the patient improves on continuous insulin infusion and blood glucose control, as did our patient [5,7-9].

It is therefore beneficial to include HHNS and new-onset diabetes mellitus in one’s initial list of differential diagnoses in a patient presenting with a focal seizure and hyperglycemia [8,9]. Our case is unique, in that our patient was of advanced age of 85 years and had never been diagnosed with diabetes until he had a simple partial seizure of the left wrist. Had our index of suspicion for HHNS-induced seizure been lower, our approach would have been to focus on and treat the seizure, which would not have benefited the patient, and instead may have sedated him further.

## Conclusions

HHNS is a severe, potentially lethal complication of diabetes mellitus type 2. In undiagnosed diabetic individuals, HHNS can present as neurological chief complaints, including the presence of focal seizures. Identification of focal seizures in the setting of hyperglycemia should raise the suspicion of diabetic HHNS as the etiology of seizure activity and should be managed accordingly, with an insulin infusion and initiation of outpatient diabetes management.
